# Managing racism? Race equality and decolonial educational futures

**DOI:** 10.1111/1468-4446.12976

**Published:** 2022-09-06

**Authors:** Suki Ali

**Affiliations:** ^1^ Department of Sociology LSE London UK

**Keywords:** anti‐racism, audit, decolonial, education, feminist

## Abstract

The Office for Students is now holding UK universities to account for their failures to address racial inequalities, and the Teaching Excellence Framework is bringing the student experience to the fore in assessing higher education institutions. Racial inequalities persist in spite of decades of legislation aiming to promote equality and end discrimination. The paper considers two main areas of racial equalities work, namely, (1) anti‐racist and (2) decolonial initiatives. It suggests that the rise of managerialism and in particular, audit cultures, have allowed racism to flourish in spite, or because of, the need to account for equality, diversity and inclusion in global markets for higher education. Auditing requires a focus on identities, and cannot take into account the complex ways in which race, race thinking and racism are maintained in knowledge production. The lack of consensus around what decolonial education should be undermines attempts to produce educational social justice. From a feminist postcolonial perspective, the paper suggests that recentralizing racism and reengaging difference offer an important way to negotiate more just educational futures.

## 1 INTRODUCTION

In the past five years, UK Higher Education Institutions (HEIs) have been under increasing public scrutiny for failing to address racial inequalities across the sector. Under the new Higher Education and Research Act (2017), universities are required to review all aspects of the educational experience and delivery, including teaching, and will report to the recently formed Office for Students (OfS). At the launch event for the OfS in February 2018 Sam Gyimah (former Conservative Universities Minister) heralded this “new” era, with its new regulatory controls, by delivering a speech entitled “A Revolution in Accountability” (Gyimah, 2018). The urgency behind such activities appear to show a newfound political energy to improve access to and outcomes in universities, and a social and political concern to advance “race equality” in the UK (e.g., The Race Disparity Audit, 2017/2018). This “top down” concern emerges alongside a more vocal and visible set of student demands and campaigns for educational (racial) justice. In the same speech, Gyimah candidly acknowledged that “Not a single week goes by without a university story being splashed on the front pages.” (Gyimah, 2018). However, of course, racial inequalities are not new, campaigns for equalities are not new, and legislative and policy interventions are not new. Indeed, these changes are taking place some 40 years after legislation to end discrimination was passed in The Race Relations Act (1976) with further Acts following in 2000 and the Equalities Act of 2010. In addition, in 2016, the Equality Challenge Unit, now Advance HE introduced a race equality charter mark (REC) which has targeted inequalities for BAME^1^ staff and students in H.E.

This paper will explore the consistent failure of HEIs in England^2^ to significantly advance “racial and ethnic” equalities (Advance HE's terminology). It follows and draws from a long line of work that has engaged issues of racism and inequality in universities (e.g., Ahmed, 2012; Alexander & Arday, 2015; Bhopal & Pitkin, 2018; Johnson et al., 2018; Sian, 2019; Tate & Bagguley, 2016) to argue that the problem is the refusal to take seriously the persistent, long standing disadvantages that BAME people face as a result of differing, interdependent forms of racism within universities—and wider societies. To explore this, I examine two key approaches to “the problem of race” in the academy: Anti‐racist (AR) initiatives and decolonial (DC) initiatives. I start from the premise that these are fields of politics and knowledge that are unstable and contested, and that these characteristics underpin a lack of clarity in usage, and thus their implications for HE. I argue that though the two initiatives are not discrete entities, they currently engage somewhat different approaches to similar questions, focusing on different aspects of educational experiences—that is, the individual as actor in the academy and systems of knowledge production. Both are heavily enmeshed in ideas about the impact of and potential for (racialized/ing) identities and subjectivities in education and wider society. The focus on identities, while legitimate and understandable ‐ perhaps even essential ‐ can have a limiting effect on radical change given the different ways in which race and ethnicity are experienced and understood across time and place. However, identities remain important loci for intervention with political attacks on group and individual positionalities at a time of resurgent populism, nationalism, xenophobia and racism, not only in the UK in the post‐Brexit era, but across the globe. Contemporary British tensions about race and nation have their roots in the longer political economy of race, racism and imperialism, and fears about sovereignty in light of European federalism (see Virdee & McGeever, 2018), and are exemplary of the continuing present of racializing, colonial thinking in neoliberal, marketized, managerialist higher education.^3^

In such a reactionary and conservative political climate, it is especially important to centralize the issue of racism through an analysis of flows of power in academic knowledge production, while recognizing the diverse ways in which race and ethnicity are understood in different contexts around the globe. I argue that the major challenge facing both initiatives is the continued commitment to “manage racial inequalities” out of HE. Much of the work that is being done takes the form of “tinkering” at the edges of the problem via the core modes of governance and regulation within university “audit cultures” (Power, 1997; Strathern (ed.) 2000), and necessarily remains firmly situated in a neoliberal model of HE which requires and therefore maintains hierarchical difference. Education is big business and as Gyimah proudly notes “[I]n almost every international league table, we (Britain) are a global superpower in HE, second only to the US. The brightest and the best from around the world are queuing up to study here.” (Gyimah, 2018). I suggest that current initiatives are (still) largely aimed at incorporation of difference, often through Equality, Diversity and Inclusivity agendas, into a neoliberal model of success in competitive markets. In light of the failures of this approach to tackle discrimination, there needs to be renewed urgent, energetic, and honest debate about the fundamental questions about what and who HE is for. We, that is, academics, professional staff and students, need to properly acknowledge and debate the contradictory views on this that are so evident in contemporary universities. These dissenting and often hostile positions are found in all areas of educational experience—across politics, epistemology, policy and practice, in recruitment, educational design and delivery and social and spatial environments. It is only by trying to negotiate with these differences that we will begin to develop shared strategies for challenging racism, and developing radical and liberatory education.

I begin by setting out some of the context and background to the current initiatives. I go on to outline some key features of anti‐racist education and campaigns to decolonize the university—and the curriculum in particular. To analyze this complex issue, I use a range of methods from across a range of sites. In this paper I draw on over 10 years' experience of working on racial and ethnic equality issues and policy in a Russell Group university. This is supplemented by further informal and formal data gathering through public race equality networks and sector events over the past 20 years. As such the paper draws on auto/ethnographic material, and I also use data from a small‐scale qualitative project about developing and sustaining an “inclusive curriculum” within a so‐called “elite” institution. In short, this is a paper that draws upon materials and data that come from both within a single institution and across the sector over the large part of working life as a woman of color who has experienced much of which I analyze here. For this reason, to protect anonymity, I deliberately obscure detail about which institutions, when data was gathered and detail on who is speaking and use representative quotes and observations that illustrate patterns and themes under investigation. For the purposes of this paper I draw more heavily upon work with and for students than staff to consider what is at stake in the shift in discourse away from anti‐racist work to decolonial work in universities. As suggested above, it is evident that both approaches raise important questions about the role and importance of identity, locatedness and positionality in knowledge production within and outside of HEIs. More importantly, they reveal the ongoing instability of the term ‘race’, the importance of racial knowledge and the tenacity of racisms. I argue that understandings of anti‐racist and decolonial work often do not share many of the aims and objectives of “equalities agendas” which have come under the control of managerialist, audit cultures and the discourse of accountability. I suggest that there appears to be a political and intellectual amnesia about earlier critical interventions on race, racism and ethnicity; and that this and contemporary critical work can challenge the corporate approach to equalities in universities as businesses.

### 1.1 Contextualizing educational change

Since its beginnings, formal barriers to higher education in the UK ensured it was largely the domain of elite, upper class white men (Palfreyman & Temple, 2017). In the post‐discrimination legislation era, during the 1970s and 1980s, more systematic attention turned to failures of universities in attracting and providing for minoritized groups.^4^ Despite the expansion of fees under the Labor Party in 1998, and their increase under the Conservative‐Liberal Democrat coalition in 2012, HE has continued to expand, and numbers of BAME students entering HE is increasing year on year, outpacing the rate of their white counterparts (Advance HE, 2019b). This is seen as great progress given that in the past educational inequalities and discriminatory practices had resulted in lower proportions of BAME students entering universities than that of white students. The term “BAME” is problematic in itself, but also often hides another important dimension to the statistics which is how many of the students are UK domiciled or “home” students and how many are from other EU countries or other non‐EU international backgrounds. For example, summary statistics released by the government in June 2019 are based upon “home” students. While they show that all racial and ethnic groups are entering HE at higher rates than ever before, a closer analysis shows the highest rate is among Chinese students, while in the section on degree classification, the category “Chinese” disappears and we can only presume is subsumed into the “Asian” category (Ethnicity Facts and Figures, 2019). These kinds of differences are crucial as we follow the statistics from entry to outcome and exit. Here the picture is not one of unequivocal “success”. Simply getting minoritized students in to HE is a beginning. The promise of HE is one that remains unfulfilled for many. Figures show that BAME students face an “attainment” gap in outcomes (Advance HE, 2019b, their term), are more likely to drop out, and are encountering high levels of harassment (EHRC, 2019). Yet again, the details matter. The “attainment” gap, more accurately understood as an awarding gap, between white and Black students (their labels) attaining a first/2:1 is 24.6% points, with Black African at 23.9% points and Black Caribbean at 21.7% points, while figures for other racial and ethnic groups are lower for example, for Chinese students the figure is 4.3% points (Advance HE, 2019b). The obvious questions are why are these inequalities persisting, and what can we do to eradicate them? In the past “deficit models” peddled the myths of lack of aspiration, application and ability of BAME people, but we are now told that this is no longer the case.

At a session on educational delivery at an HEI in England in 2017, figures were presented about the “attainment” gap for Black students. It became clear there was some ongoing conflation of Black “home” students with working class‐ness, “disadvantage” and educational deficit. I raised a concern that this unconsidered conflation was over‐simplifying a range of issues, homogenizing a wide group, while also sounding perilously close to a newer kind of deficit model based in “common‐sense racism”; one which suggests that it is not the intrinsic and innate inability (intelligence, potential to learn) of BAME students that is the problem, but rather their “background” is the problem for the institution to solve. This is still perceived as a problem that lies outside of the university and is characterized as a property of the individual and a diverse group. Not 5 mins after I raised this concern, a questioner asked

‘Is this because the black students are poor?’

(White, male northern European academic)

This comment reveals not only in the way my intervention was erased but is a stark reminder of the ways in which these common‐sense discourses that stereotype entire groups are so powerful and resilient. However, the uncritical conflation of “home” BAME students with deprivation and “pipeline” problems resulting from secondary education, while stereotyping on one level, is based in some fact given the wider racial educational and societal inequalities in Britain. It is often the case that working class BAME students from schools that are struggling will have experienced less effective secondary education, have faced similar (racist) erasures and assumptions about their suitability for higher education in their teaching and learning, and had to deal with more overt racism and discrimination (see e.g., Gillborn, 2008). Further, they may also be dealing with the ongoing effects of racism and discrimination against them and their families in their wider lives.

Drilling down behind the statistics and drawing upon qualitative methodologies to supplement the bald figures reveals that universities are often hostile environments for BAME staff and students, and that they face discrimination and erasure at all stages of their careers. A recent Equalities and Human Rights Commission (EHRC) report into racial harassment in HE shows that BAME students experience high levels of harassment and even violence (EHRC, 2019), as do staff (see e.g., Alexander & Arday, 2015; Bhopal, 2016; Gabriel and Tate 2017; Johnson et al. (eds.) 2018; Mirza, 2018; Sian, 2019). These kinds of accounts are reflected in these very visible stories in the press that Gyimah (2018) refers to above. Perhaps universities are finally being forced to recognize that it is not a stereotypical or even collectively stereotyping view of a highly diverse group that is the problem; it is the failure of the sector to address structural, cultural, policy, practice and process disadvantaging of BAME staff and students.

The picture then might generously be described as “mixed”. While it is hailed as a positive that more BAME students are entering HE, their experiences and outcomes are often more negative. It is also clear that BAME students are unevenly spread across institutions and across disciplines and courses. HESA statistics show that more BAME students are in STEM subjects and many more are in newer universities than are in the so‐called Russell Group of Universities (HESA, 2019). Year on year we have been assailed by headlines naming and shaming universities for the “lack of diversity” in their intakes. Again, the undifferentiated term “BAME” hides the fact that entry levels for Asian and mixed‐race students are higher and Black students significantly lower (HESA, 2019).^5^ This uneven intake is only exacerbated by higher rates of drop out, and lower outcomes for these students across the sector.

AR and DC agendas are aimed at challenging racist and colonial education. However, anti‐racist work in academia, once linked to activism in wider society, has become subsumed within the “equalities” agenda. Decolonial agendas, originally global or transnational student‐led movements, have also increasingly been co‐opted into “equality, diversity and inclusivity” work, and in turn, used in institutional strategies for branding and market share. As long as a disconnect remains between the intellectual, political and moral understandings of race, racism and ethnicity and the regulatory, managerialist approaches to equalities, we will not eradicate racial injustice in education.

## 2 INEQUALITIES AND ANTI‐RACIST UNIVERSITIES

One of the most significant moments that still underpins much of the work on anti‐racism is the introduction of the Race Relations Act (“RRA”) in 1976.^6^ This Act is notable for being the first to provide extensive directives about education. Discrimination on the basis of “…. color, race, nationality or ethnic or national origins” were made unlawful. Discrimination was defined as treating people “less favorably” on “racial grounds”, or where an institution or body applies a “requirement or condition” that isn't required of others; is not justifiable irrespective of “racial origins”; and finally “…. [I]s to the detriment of that other” because they cannot comply with it (Race Relations Act, 1976). The Act underwent further amendment in 2000, again specifying race equality in education. The Equality Act 2010 required institutions to shift from dealing with discriminatory practice retrospectively, to demonstrating proactive promotion of equality for all those with “protected characteristics” and an imperative to address “socioeconomic inequalities”. The newly defined characteristics no longer mentions “color” but retain ethnic group and nationality. These developments reflect changes in understandings of race, ethnicity and racism alongside a visible anti‐racist movement that involved both popular culture and scholar activists writing about race, ethnicity and racism in the 1970s and 80s (Bhattacharyya et al., 2020; Brah, 1999; CCCS, 1982; Gilroy, 1987). The Equalities Act also now includes “faith and religious belief”, broadening previous provision in the RRA prohibiting discrimination against Sikhs and Muslims. This is too demonstrates how histories of colonialism, imperialism and migration and settlement lead to situated racial formations in national contexts.

It is arguably the Macpherson Report that continues to have most impact in the day‐to‐day discussions about race equality in organisations such HEIs. MacPherson centralized racism, rather than discrimination or hatred (though both of these are important). The emphasis on structural or institutional racism reframed the problem away from individualizing, psychologizing, occasional “bad apples” holding outdated and offensive beliefs, to focusing upon organisations as a whole disadvantaging racialized minorities. Macpherson defines institutional racism as:

The collective failure of an organisation to provide an appropriate and professional service to people because of their color, culture, or ethnic origin. It can be seen or detected in processes, attitudes and behavior which amount to discrimination through unwitting prejudice, ignorance, thoughtlessness and racist stereotyping which disadvantage minority ethnic people.

(MacPherson 6, p. 34)

Macpherson coupled with the Equalities Act should then provide a strong framework for making racism more visible, for holding institutions to account, and for insisting that there should be consequences for racism impacting staff or students.

In the wake of MacPherson's report, a raft of “anti‐racist” training was rolled out across a range of public institutions. The purpose of the training was to raise awareness of the pervasiveness of racism, and its detrimental effects on individuals, and, most importantly, to challenge racist behaviors. It was not long before research began to suggest that those most likely to need this training were often alienated by such direct approaches and they were not effective. In summary, it was noted that the training could result in people feeling that they couldn't express views or raise questions and concerns about race and racism for fear of saying the wrong thing. It was feared that those who might have held racist views simply chose not to express them in these settings, driving racism undercover. AR training might then not effectively challenge racist belief systems and structures of privilege that supported them (see e.g., Bhavnani et al., 2005).

The perceived harshness in forcing (white) people to confront their complicity with racism was mirrored in other debates about, for example, defining and tackling sexism and misogyny. The 1990s, which were characterized by extensive discussions about in/equalities, shifted discourses away from equality of opportunity to equality of outcome. In gender and disability terms, it was argued that the aim was not to treat people “the same” as they are not on a “level playing field”. As social and educational histories often resulted in pupils and students starting their education from very different places, therefore to treat them as if they were in the same place was advantaging those who already had the resources to navigate systems confidently, actively disadvantaging those who did not. In order to better reflect this approach, and to tackle stigmatizing discourses that associated “difference” with inferiority and intrinsic disadvantage, the language of difference and inequality shifted to a discourse of diversity into the 2000s.

The move to “diversity” coincided with the so‐called neoliberalism and marketization of education, along with an increasing internationalization of HE. It has been widely noted that this corporate style of management coincided with the flattening and depoliticization of discourses on all kinds of discrimination (e.g., Ahmed, 2012). While terms such as neoliberal, market and internationalization are imprecize and contested, they nevertheless describe the expansion of a global market in education, the expansion of fees and other funding changes, and the requirement of universities to be savvy and profitable businesses. These changes undergird the shift to market speak about difference and diversity (Ahmed, 2012). The marketization of HE begun in earnest under Margaret Thatcher's Conservative government and continued with the rise of New Labor, culminating with the Conservative‐Liberal Democratic coalition changing structures of funding in HE. “Valuing diversity” in market terms has become a dominant mode for persuading universities to take action. Simply put, diversification and inclusivity (of the right kind) mean greater success and better profitability for the institution.

Within this context, while identities provide a source for knowledge and solidarity, they have also become a site of institutionalized, regulatory compliance. In the wider socio‐political climate at the end of the 1980s and 90s through to the 21st century, identity was at the heart of British (race) politics with integration’ of minorities and “accommodations” for those from ethnic or religious minorities central to debates about the effects of multiculturalism on Britishness. Race was at the heart of these debates, though often hidden in discussions about promoting “British values” and behaviors (Ali, 2014).

### 2.1 From anti‐racism to diversity and inclusivity

The transformation of HE in UK to “market based” approaches to tertiary educational provision, and the foregrounding of international marketplace and the global competition for students underpins the move away from a focus on discrimination and racism and onto the development of “equality, diversity and inclusivity” agendas.

The loss of the critical edge in anti‐discrimination work has been written about extensively and it should be noted again that these are not new concerns or arguments. By the late 1990s, critical educational research in all sectors raised concerns as to how the new discourses impacted in and on practice. For example, in her work with children with “special educational needs” in secondary schools (a term she describes as “uncomfortable”), Shereen Benjamin asked

Is `valuing diversity’ on its way to becoming a cliché: nothing but a euphemism for the enduring reproduction of oppressive social relations and consequent material inequalities?

Benjamin, 2002, p. 310

The way in which the cliché works to empty the meaning and impact of (hierarchical) differences in HEIs is through a teleological argument that because the inclusive university is diverse, that diversity means that the university must be inclusive. Being inclusive means that no one is being excluded, therefore they are not being discriminated against, and thus the university is demonstrably a place where all is fair and equal. Sadly, this simple equation is evidently untrue, as it does not result in equality or an end to racism as the figures above show. Instead, it shifts the language to a neutral, apolitical and largely apologist agenda. As universities state their intentions to be inclusive and diverse environments, their statements stand in for action and the non‐performative effects mask inaction, or worse, the refusal to engage with demonstrable inequality (c.f. Ahmed, 2007). If you are narrating your evident inclusivity, it must necessarily be very perplexing as to why some people are doing badly.

It is no surprise to see that this kind of language central to Unconscious Bias (UB) training in HEIs. The move from “racism” to “bias” is significant. In one UB training session I observed, a case was used of a senior white, male, academic who found it hard to remember the Chinese students' names, and who just accepted that he would never learn them. A participant questioned if this was “racism” rather than unconscious bias, and the trainer stated categorically that it was not racism as the Professor “didn't know” that what they said was potentially problematic. Setting aside how it is an educator in a university may not have realized this was problematic, the issue at stake was intentionality. Here Macpherson's use of Scarman's concept of “unwitting racism” (Macpherson, 1999, p. 6.15) might be appropriate, but still leaves the question of how to mitigate against such behaviors and beliefs unanswered.

This failure to engage with the structures and cultures that support such views or to challenge them has led to widespread concern with UB training, even from within corporate organisational sites. There is increasing evidence that UB training can worsen bias (EHRC, 2018), or as one researcher from Chartered Institute for Personnel Development (CIPD) suggested, can “unleash it” (CIPD, 2019; Palmer, 2019), by letting people off the hook through the perspective that if they don't *intend* to be biased it is all OK. This formal sector and policy research confirms what many of us have been experiencing and speaking out about, and scholars have written and published on for some time (e.g., Ahmed, 2004; Ahmed, 2012; Noon, 2018; Tate & Page, 2018). Indeed, we might return to Fanon who stated:

For a time it looked as though racism had disappeared. This soul‐soothing, unreal impression was simply the consequence of the evolution of forms of exploitation. Psychologists spoke of a prejudice having become unconscious. The truth is that the rigor of the system made the daily affirmation of a superiority superfluous.

Fanon, 1964, p. 38

Universities have plowed on with a system that is ineffective and doesn't engage with the multiple forms of racisms—structural, institutional, cultural, interpersonal, direct, indirect, overt, covert etc. ‐ that result in serious adversity for BAME staff and students. Worse, it provides an inoculation for those in power against accusations of indifference, inaction or hubris. The recent EHRC report into “racial harassment” records “anti‐white bias” alongside and as if it were equivalent to racism against BAME and students of color. Yet taking this position is seen as a way to “open conversation”, and get people “to the table” or “to buy‐in” (phrases I have heard used repeatedly). In a climate of permissive forgiveness, people slide from plausible deniability, often couched in terms of ignorance, to disavowal. A major problem facing BAME staff and students is the outright denial of the frequency and effects of racism when they speak out against it or point it out in processes or practices (Batty, 2019).

This is the climate in which the anti‐racist work takes place, where people are afraid to talk about race and racism, unless of course they are the ones on the receiving end of it and live it. Nonetheless, universities are compelled to address diversity and inclusivity in a “global knowledge economy” and an international market for students and staff.

For example:

A PhD student observed that her supervisors kept pushing her to do work on China. She stated that it was very hard to resist them but she wanted to work on American imperialism. … Interestingly she suggested it was a form of subjectivation, whereby she was being subjected to become a Chinese student.

Notes from a Decolonial event 2019

The implication here is that a student was accepted with a research proposal in progress, but after arrival, this became problematic. This could be a further aspect of global market competition for students. The student's concern can also be read as a stereotypical gendered racism at play as the “subjectivated” identity coincided with performing an appropriately grateful, female East Asian woman. I have heard many stories of gendered, sexualized racism from other young women students who may not name their experience “racism” per se due to their complex situated experiences and political and intellectual perspectives on discrimination and “bias”.

The benefits of anti‐racist approaches are that they keep focus on racism itself, on its pernicious and ongoing impact in HE, and on the ways in which individuals can be complicit with or challenge behaviors and structures *regardless of their own positionality*. In removing anti‐racist discourses, we leave HEIs and importantly, the people who work in them, free from accountability, and allow them to mask continuing discriminatory practice in the language of inclusion and plurality. And while “white fragility” remains largely unaddressed in HEIs (DiAngelo, 2011), awareness of the symbolic and structural power of whiteness is more evident in research in UK education (Bhopal, 2018; Tate & Page, 2018).

Insisting on facing and eradicating racism allows us to challenge those who spout platitudes about “diversity and inclusion” while allowing racist processes, practices and outcomes to flourish and expand. The harshness of the term reflects the need to address the harshness of experiences of BAME people in HE. This squeamishness about using the “r” word is hampering progress to truly liberating education not only for “social justice” but also the recognition that access to radical education itself is an issue that underpins and *is* social justice. Rather than agreeing with claims that in the post‐race era there is racism without race, I would argue there is a return to race talk in HE, and with it race thinking, but without the language of racism. We are in the curious position of having clear evidence of experiential and structural racism, but now it is not due to “color‐blindness” or “racism without racists” (Bonilla‐Silva, 2014) per se. We now have no racists *and* no racism ‐ just people who have natural, unconscious bias and regulated, diverse and inclusive institutions. These positions are justified through discourses that only focus on “inequalities” as organisational and pedagogic failures. So how might decolonization work differently than this? Is it an inevitable outcome of the failures of the anti‐racist approaches, or their occlusion and omission by the diversity agenda?

## 3 DECOLONIZING UNIVERSITIES

The move to decolonization in UK educational discourse arises at a time that the international market in education has been at the forefront of concerns in UK universities, and it is significant in that it has been largely student led. Emerging as it did in the Global South, and with particularly influential campaigns coming from South Africa, Latin America and calls for indigenous knowledge recognition in Australia and New Zealand, the decolonization agenda appears at first glance to be significantly wider than that of anti‐racist initiatives that focus on “bias”. In the South African context, decolonizing education was not some kind of “diversity exercise”, but South African scholars' attempts to liberate themselves from the tyranny of colonial education (Heleta, 2016). Some early student campaigns focused upon the symbolic legacies of colonial oppression that have dominated the educational spaces of South African campuses and also include protests against fees. The UK has seen these concerns echoed with the “Rhodes Must Fall”, “Why is my Professor not Black”, and “Why is my Curriculum So White” campaigns.^7^ But the key thinking behind the decolonial initiatives is that the colonial education system has been nothing less than an ongoing colonization of the mind, of thought and of the imagination and therefore of identity itself. For a number of scholars (who may have been previously described as or differ from those designated “postcolonial”) there can never be true liberation ‐ political, intellectual, or cultural ‐ while the colonizers' language and education is imposed upon the colonized. These ideas put the context and structures of learning at the heart of the liberatory project but also centralize the role of epistemology and ontology and the exclusions of the category of ‘the human’, as discussed below.

While it is not possible nor desirable to “list” all influential thinkers that come from across the Global South, it is important to note regional variation in thinking and debates on temporalities, terminologies and agendas (e.g., “waves” of postcolonial thought Go, 2016), and that even this narrative is itself contested (Bhambra, 2014b). As Bhambra et al. note “ … decolonizing” remains a contested term, consisting of a heterogeneity of viewpoints, approaches, political projects and normative concerns' (Bhambra et al., 2018, p. 2). Some shared issues in the global North however include a concern to historicize and provincialize Anglo‐European knowledge production from the metropolitan centers (Bhambra et al., 2018, p. 3). This strategy has been discussed in sociological terms as challenges to imperial or colonial epistemes (Go, 2020; Meghji, 2021).

As Connell has argued “Contemporary universities are powerful institutions, interlinked on a global scale; but they embed a narrow knowledge system that reflects and reproduces social inequalities on a global scale.” (Connell, 2017, p. 10). Decolonial and postcolonial scholars argue for a reckoning with histories of the present in social theory and outline multiple ways to achieve this through perspectival realism or postcolonial social theory (Go, 2016, 2020); the valorization of Southern theory (Connell, 2007, 2017); creative synergies of theory and decolonial sociology (Meghji, 2020, 2021) or relational or connected sociologies (Bhambra, 2014a).^8^

For many authors, the decolonial project is nothing less than the rehumanization of entire swathes of people who have been rendered less than human in order to exploit and control them. For a number of these scholars, knowledge is embodied in subjects who are located in particular times, places and spaces, and in order to thrive, universities must completely transform (e.g., Mignolo, 2009; Wynter, 2003). This transformation must take into account the global economy in knowledge production and the material practices that support a center/periphery, metropole/colony divide.^9^ For Mignolo, “epistemic disobedience” requires a de‐linking from existing forms of Western knowledge production, and he argues

… it is not enough to change the content of the conversation, [that] it is of the essence to change the *terms* of the conversation. As far as controversies and interpretations remain within the same rules of the game (terms of the conversation), the control of knowledge is not called into question.

(Mignolo, 2009, p. 4)

So, it is evident that some crucial aspects of decolonialism diverge from those in anti‐racist work. In the next section 1 consider why this is by looking at the socio‐political context in which decolonization comes to the fore in English educational discourse and institutional contexts.

At this moment, the challenges to HE that have come about by massive changes to structures and funding for both universities and students are shaping the engagement with “the market”. It is beyond the scope of this paper to unpack terms such as “neoliberalism” and “the market” in any great depth. Many educators and practitioners are using the terms in imprecize ways themselves, but with a shared sense of what this means for them; including the imposition of economic value systems onto education and knowledge itself, the impact of a global marketplace for education on university funding and provision, a quantification and commodification of learning, and an emphasis on the student as consumer who “invests” in a degree to reap future earnings (e.g., Collini, 2017). The overt commodification of knowledge is a double‐edged sword. In some ways, the hike in fees, global visibility of statistics on outcomes, teaching quality, staff‐student ratios and so on, position the student‐consumer as sovereign and have therefore given students a further “power” that they may not have had in many decades. Universities are afraid of students voting with their feet—and their wallets. The new Teaching Excellence Framework (TEF) places “student satisfaction” at the heart of the educational offer such that some academics have expressed concern over the metrification and quantification of learning experiences that will force academics to respond in ways that may not be commensurate with wider teaching aims (see e.g., Campbell, 2016). The student led nature of the decolonial campaigns becomes absolutely crucial and wields some influence, though the visibility of these campaigns largely centers on bigger and Russell Group universities where “international” means global league tables. Engagement with the Global South is now also a visible marker of internationalization and diversity.

Student demands are wide reaching and also question universities' investment choices and structuring neocolonial relations of power not only in knowledge production but in business choices. Elite universities are investing millions in global markets and students (and staff) are concerned that this investment should be ethical,^10^ while others are being challenged on promises made in marketing designed to attract students (Bradley, 2018). This aspect of decolonization work has been less visible than the campaigns about the naming of space, colonial artifacts and monuments, and more recently in the UK, both “the curriculum” and the dearth of representation of scholars who are from the Global South, or are racialized as Black or People of Color (POC). As noted above, it appears the intended outcomes for decolonization movements are many and varied and by no means consistent across groups and locations (Bhambra et al., 2018). Regardless, they are challenging for the neoliberal university with its need to quantify both its failures and successes in student provision. For example, reasons for “gaps” in “attainment” are potentially wide ranging and requiring situated analysis in HEIs. Yet decolonial discourse has been used in curriculum initiatives, and in ways that may exacerbate rather than address limiting race thinking as I explore below.

### 3.1 Reading lists and representations

Just as anti‐racist work has been diluted by the diversity agenda, so too decolonization activities have struggled to maintain credibility and effectiveness once university‐led EDI initiatives take them over. In much of the literature universities make available online, the inclusivity and diversity agendas and decolonization agenda often appear to coincide most obviously in the arena of the curriculum. While inclusive curriculum work has its roots in disabilities activism (Gibson, 2015), it is now often synonymous with the decolonization agenda.^11^ However, decolonizing the curriculum must engage with every aspect of design, delivery and assessment. This includes vexing questions not just on who is teaching, but on how and what they are teaching. In the UK, the related campaigns “Why is my Professor not Black?” and “Why is my curriculum so white?” both address these issues as a pair which are part of the same problem. Yet this simplified approach to challenging the white‐centrism of much of the work in the social sciences has also been fraught with frustrations and compromises, and can still mobilize identity and positionality in problematic ways.

It is not helpful to increase citations of women and minoritized scholars (as if the two were always only ever separate), and to incorporate linguistic and geographic variety in readings if the way in which this work is taught retains the core/periphery, canon/critique model of teaching delivered by disinterested academics, or some who might even be hostile to the work of the subaltern scholars that they are teaching. There is also the ongoing concern with “academic freedom” which resulted in one white male professor suggesting that what he was being asked to do (review his reading lists) was “Stalinist”. The lack of BAME academics may shock university leaders ‐ only 0.6% of Professors in the UK are Black and there are disproportionately low figures for other minoritized groups ‐ but the pipeline into academia is leaking, with high rates of attrition for BAME students (Advance HE, 2019b). A cynical belief in meritocratic systems, and the myth of the obviousness of academic “quality” can explain why both representation on lists and in jobs, is slow to change. As social scientists we should know that what constitutes “good” work in any given discipline is socially produced and maintained in ways that allow universities to reproduce themselves in their own images. This social shaping and transmission of “quality” is also a movable feast, allowing hiring and promotions committees to bend rules, regulations and policies to breaking point. The narrative that “the best person for the job” will always get it is extraordinarily flexible. Best for whom? Best at what? Best for what purpose? By what measures?

It also bears repeating that many of these discussions essentialize the characteristics of minoritized scholars in the crudest of ways. Not all BAME scholars are doing (critical) work that engages theories and perspectives from the Global South. Indeed, it would appear those that succeed in HE must, to some extent, conform to the universities' expectations of them, so it is likely that someone who believes in the meritocracy themselves, who can speak to the “EDI agendas” in supportive ways, is more likely to get the job. For those who cannot and will not “play the game” the outcomes are often harsh, and unconscious bias has little to do with that. It is the shape‐shifting nature of racism and discrimination that succeeds in excluding many, while graciously including those who are closer to the dominant ideals. How could it be otherwise in the era of the Charter Mark? And is this “what students want”? The answer is some but not all as this quote on representation in staffing illustrates:

XXXX [a student] noted that it was difficult to hire professors for their viewpoint and academic history and be diverse at the same time. He noted that whilst the curriculum is overwhelmingly white, he would rather that it was white than people trying to teach things they don't understand. In particular, that he would rather have a graduate teaching assistant who provides good feedback and is able to speak clearly, but it doesn't matter to him if they are from the same ethnicity as him. As a Chinese person he does not care, because he is driven by other things.

XXXX responded by stating that of course that should be a minimum expectation but that there were additional benefits to ensuring that staff were also representative of the student population.

Official notes from Workshop, Race in H.E. 2016

The same questions pertain to the content of the curriculum. There are potentially as many BAME and PoC scholars across the globe who hold racist, sexist, homophobic, transphobic, ableist etc. views, as there are in the rest of the population—in spite of appeals to cultural values that challenge hierarchizing or exclusionary views. Decolonial programmes that tinker with reading lists should take heed of feminist debates about the “mainstreaming” of work on racialized gender, who teaches it and what this means for “intersectional” gender politics (Ali, 2009). There is no consensus on what constitutes, feminist work, nor yet what its purpose is, and plenty of it falls squarely within the most (“race‐blind” or white‐centric) neoliberal version of equality (see e.g., Banet‐Weiser et al., 2020; Fraser, 2016). I have observed, as have others, that the “tinkering with the reading lists” approach is having a similar effect to UB training. It can allow academics or departments or institutions to suggest that they are “doing something” about inequalities through representation, without addressing the major epistemological and pedagogical challenges they face.^12^

The decolonial agenda is open to as much corporatization as any other aspect of radical and transformational pedagogies and epistemologies. A further problem is the lack of consensus on what a decolonized curriculum or university might look like. Below I explore some of the most influential approaches to decolonizing education and the implications for “race thinking” in HEIs.

## 4 DOING ANTI‐RACIST AND DECOLONIAL EDUCATION

The argument proposes that the struggle of our new millennium will be one between the ongoing imperative of securing the well‐being of our present ethnoclass (i.e. Western bourgeois) conception of the human, Man, which overrepresents itself as if it were the human itself, and that of securing the well‐being, and therefore the full cognitive and behavioural autonomy of the human species itself/ourselves.

(Wynter, 2003, p. 259)

I have outlined key aspects of anti‐racism and decolonisalism above to show that there are continuities in focus and practice, and also areas that might be seen to diverge. Of course, it is possible to say that decolonial work has to be anti‐racist, and anti‐racist education has to be decolonized. In reviewing these areas, I am not suggesting that they have totally distinct trajectories but rather I am exploring why and how attempts to reduce inequalities have failed, and where they may be successful. Decolonization may not necessarily be considered to be about “race” per se, or even racism, if this is not the language nor theory used to explore hierarchical relations of power. For example, a decolonization project in Eastern Europe engaging with situated spatio‐temporal colonial relations of power, may draw upon somewhat different approaches to those used by scholars and students in post‐European imperialism and colonialism in India, which has been shaped by other specific ethno/religious/caste/linguistic affiliations and struggles. But we might say that race and racism can or should be central if it refers to European colonial and Imperial powers' interests in the global south with non‐white Others, as well as “settler” colonial societies in the global north such as Australia, New Zealand, the USA and Canada. English knowledge of the colonial “Other” was produced in dialogic relationship between metropole and colony, founding a form of “modern” race thinking based upon “scientific” authorization, and that raciology underpins coloniality/modernity to the present (Quijano, 2007). For some, by extension, whiteness is at the heart of universities' knowledge production, yet others who may agree with this in principle, believe we need to take care not to erase the complexity of the production and maintenance of whitenesses, and exclusionary differences.

The following quote is extracted from a discussion at which several Black postgraduate students recounted their experiences of a course that engaged with colonialism in Africa.

‘It is not about race, it’s about epistemology’

East African student to African American students

All the students had concerns about their teaching. The African American students made a strong case that the comments expressed by a lecturer on the “benefits” of colonialism were racist. While not negating their perspective, the above comment was made by one of the East African students. This exchange throws some of these issues into sharp relief. This discussion progressed productively as they expanded on their slightly differing perspectives on the problem, but their similar suggestions for potential solutions. These included ensuring suitable hires of Black African scholars, with regional expertize, who could more credibly and perhaps, thoughtfully and ethically, teach on legacies of African colonialism and their ongoing impact. As Andrews suggests this is a two way process “ … if you decolonize your knowledge base you will quickly decolonize your staffing” (Andrews, 2018, p. 132).

We cannot, of course, assume students of color hold identical political and intellectual positions, or that they share inevitable racial and ethnic solidarities. The students above shared their basic knowledge of and concern with a form of “white, Eurocentric” teaching. But we also know that many “international” students report coming to the UK to learn about Europe and European thinking that is not available to them in their country of origin. When applying to elite universities, they are hoping to gain the social and cultural capital that comes from attending such universities, as are many “home” BAME students. The inclusive curriculum project revealed this range of perspectives, including BAME students being placed into racializing “boxes”. For example, the following is an example of an experience common to many:

‘I don’t want people to assume things about me. In one course the lecturer asked if I wanted to do the week on race—they just assumed I would do that’

Black British woman, Interview with undergraduate student 2018

BAME students are constantly expected to represent “their communities” or teach others about race, and to handle the “race stuff”, regardless of their academic interests.

A number of obvious things arise here. If the “stakeholders” in universities do not share perspectives, what are “we” fighting for? Many staff and students invest time and money to gain advantage in real world scenarios of work under conditions of intense competition and increasing precarity. They are often not interested in dismantling anything so much as gaining entry into the existing structures, and in that sense “inclusivity” is what they are after. Others are simply unclear as to what these debates mean to them, and are still questioning their own relationships to knowledge production

‘If you sit on the fence and you’re not 100 % adamant on their views, and passionate about it, you feel like you can’t become involved in the discussions, because it seems like it’s quite a radical discourse.’

White British female Interview with undergraduate student 2019

This young woman's quote is typical of many for whom these issues are important, but sadly, still quite new. The contrast with the often well thought through and articulate positions that BAME students bring to analyses of their own experiences is stark, and shows how much we need sustained discussions that transcend positionalities, but also engage with the analyses of privileges or situated knowledges that preclude dialog and understanding. Current buzzwords include student participation and students as co‐producers of knowledge; here again we might look to older literature from black and postcolonial feminists on pedagogies based on experiential knowledge and non‐hierarchical learning. These purportedly participatory activities often unwittingly pull students more deeply into the service of the university as business, while putting a disproportionate burden on minoritized students to “account” for the failings of their education and provide their educators with resources and credibility while they co‐opt their work.

## 5 CONCLUSIONS: WHAT IS THE UNIVERSITY FOR? WHO IS THE UNIVERSITY FOR?

‘The fact is that what societies have wanted from their universities has been historically variable, internally contradictory, and only ever partly attainable.’

(Collini, 2017, p. 17)

A university is both a space for education and a business. In the UK that is creating tension that is coming to breaking point (Martin, 2016). Within the business model of the university, staff and students alike are increasingly (often strategically) framing their desires, expectations and experiences through the language of the market. Not only do we not share a view of what the problem is, we do not share a view on how to fix it, nor on what kind of outcome we hope to see. “We” are often not actually a recognizable “we”. In a recent published interview, Paul Gilroy (2019) discussed race, antiracism and nationalism and posed the question “what do you stand for?” rather than what do you stand against. It is an important one, and key to meaningful and lasting change. One positive initiative that has emerged comparatively recently in the UK is the first Black Studies program Andrews, (2018, 2020). Black Studies has a specific remit of education for liberation, and foregrounds heterogeneity among Black peoples and their knowledges while showing up the scale and depth of the whiteness of knowledge production in and from the global north. This important work provides space for alternative knowledges in the heart of the neoliberal university and is an attempt to “colonize” the institution from within (Andrews, 2018, p. 137). Yet Andrews writes how the program could only develop under a very specific set of conditions found in one university and is still in danger of co‐option by the university and the sector. In addition he insists, ‘Black Studies is […] not inward looking at the university; rather, it must always focus on struggles that take place outside the academy’ (Andrews, 2018, p. 140). Thus another stated aim, what is stood for, is transformation within the academy through activism and pedagogy that is in service to Black communities.

Rattansi (1997) suggests “post” in postcolonial simply indicates an ongoing and linked relationship with the colonial, rather than a time “after” when colonialism, or sexism in the case of postfeminism, are over. And at the heart of the postcolonial and Black feminist writing that has informed my work, there is of course a central refusal to perceive of the halcyon days of earlier liberal educational spaces—they did not exist (Ali, 2009).^13^ If it is true that the core of decolonial debates is the transformation of consciousness—that is reimagining the human, re‐humanizing all persons—then this cannot be done from a “western frame” alone. While embracing situated knowledges, we may want to be wary of essentializing the kinds of racialisations that perpetuate judgments on difference. The Charter Mark and Equalities agendas are firmly anchored in (British) discourses of identity, and use these in measures of success and failure. And certainly some student politics are also focused upon this aspect of the work. On many campuses debates seem to orbit around questions such as why is there not someone who looks like me, who speaks like me, thinks like me, and who re/produces knowledges that are familiar to me? Or, in another form, the question is why doesn't this education help me know who I am? These are important questions, given that for many white people this has been taken for granted. But I would also ask “why doesn't this education speak to who I/we might be/come?”. Decolonization should be centrally concerned with decentring privileged and privileging knowledges that maintain inequalities including whiteness.

While arguing that there must be space for new or multiple ways of being, many who have argued for recognition of situated knowledges are not calling for an ossification of racialized ontology. Rather, they are asking for completely new understandings of ontology and consciousness, and in this way the challenge is, what happens to whiteness? A white student who feels unable to fully grasp the politics of decolonialism undoubtedly has not really had to engage these issues in the way that many BAME students will have done, and it is too easy for the current audit work to be focused on marginal groups in ways that leave whitenesses untouched. Indeed, Purwal argues that many of the academics who are celebrated for “championing” Southern theory in the North are still embodying “somatic norms” such as white masculinities (Purwal, 2019).

Nash (2018) suggests that we need “socializing bureaucracy” to find ways of working as academics that hold us accountable for the relatively autonomous decisions we have to make. Using Weber, she argues that the “audit culture” can provide something of “stick” to balance the “carrot” of encouraging more equitable education (Nash (2018), p. 12). Yet statistics show that the legislation and Charter Marks are making slow work of change (e.g., Bhopal & Pitkin, 2018). The reports all cited above show this clearly, and we have seen that Athena Swan gender equality initiatives have failed BAME women (Advance HE, 2016; Advance HE, 2019a; Bhopal & Hendersen, 2019). Given the scope, scale and shape of racial and racist thinking there is no way that a workshop in unconscious bias, using a neuropsychological model of lizard brain inbuilt hostility to difference as its starting point, can fully engage the relations of racism, power and privilege. Courses that require us to acknowledge that “everyone is biased” but corporate social responsibility can manage this “natural” aggression to the Other, will not help us dismantle structures of inequality. However, we cannot flip the coin and refuse any of the psychosocial insights anti‐racist theorists may have.

Tinkering with reading lists will not shift epistemological hegemonies, but we also need to acknowledge that all knowledges that are deemed Eurocentric are inextricably intertwined with so called “local knowledges” (Tuhiwai Smith, 1999). Similarly, TallBear wrote that she recognized the colonial founding of anthropology, but “ … simultaneously the promise of intellectualism in helping us work our way through to another kind of world.” (TallBear, 2014, n.p). This echoes the findings of empirical work that shows how academics in the global south work with and against the hegemony of northern theory and the power of the northern academic knowledge economies (Connell et al., 2018). Andrews refers to Audre Lorde's warning that his master's tools will never dismantle his master's house. He argues that ‘[T]he struggle becomes how to subvert the tools, not how to abandon them.’ (Andrews, 2018, p. 139).

When we ask what universities are for and who they are for, we may find that there is little room for maneuver. As Bhattacharyya argues:

What fool does not understand that state‐funded institutions are unwilling to support and fund the work of revolutionary movements or to promote ideas that propose their own demise?

(Bhattacharyya, 2013, p. 1417)

It is not just the institutions; a large number of those who work and study within them share this view, with many preferring “decolonization lite” to radical reinvention (see also Dawson, 2020). An interim solution may be the compromise offered by appeals for the pluriversity. Pluriversalism engages many ideas such as Tuhiwai Smith's cited above, and would involve “…. a radical re‐founding of our ways of thinking and a transcendence of our disciplinary divisions” (Boidin et al., 2012, p. 3). The pluriverse cannot simply extend a Eurocentric model

‘ … presumed to be universal and now being reproduced almost everywhere thanks to commercial internationalism. By pluriversity, many understand a process of knowledge production that is open to epistemic diversity’

(Mbembe, 2016, p. 19)

In asking what university education is for we come up against the major fault‐lines in current practice. One aspect is that of developing intellectual capital investment, knowledge economies, employment, and research and development in globalizing capitalist markets. The other is about social transformation through self‐actualization and ontological liberation in knowledge production. These two are not compatible in marketized education. They are not equally quantifiable and not equally amenable to audit. The question is whether the audit/charter mark culture is the answer. I believe not. Marilyn Strathern suggests

‘… audit is almost impossible to criticize in principle—after all it advances values that academics generally hold dear, such as responsibility and openness about access and widening of outcomes.’

(Strathern 2000, p. 3)

If we can show, say, that of the 24% of BAME students who have been racially harassed, 20% had been physically attacked, while 56% of students who had been racially harassed had experienced racist name‐calling, insults and jokes (EHRC, 2019), the “revolution in accountability” cannot come too quickly. However, the metrics we utilize to measure change come to supplant real in‐depth engagement with the issues identified above. As Strathern puts it, “when a measure becomes a target, it ceases to be a good measure.” (Strathern cited in Collini, 2017, p. 38). Simply ensuring academics check representation in their reading lists won't bring lasting change. In much of the EDI audit work “… the result is goal displacement, where the metric means come to replace the ultimate ends that those means ought to serve” (Collini, 2017).

Holding people and institutions accountable is not enough; we need to remain critical about the form accountability takes (Strathern, 2000 op.cit.). One long line of ideas of “unintended” or “unwitting” racism and discrimination runs through the literature from the 1960s onwards and it is a “get out of jail free” card for institutions and those who work in them. It is not enough to “account for” or count inequality; responsibility needs to be taken by each individual involved in educational provision.

The Charter Marks look at both staff and students across the sector. As mentioned above, the buzzwords now often focus on “students as co‐producers” of knowledge. If we cannot encourage students to think differently about “difference” through more radical interventions, I would argue that many of these endeavors are doomed to fail. Audre Lorde argued that:

Difference must be not merely tolerated, but seen as a fund of necessary polarities between which our creativity can spark like a dialectic. Only then does the necessity for interdependency become unthreatening. Only within that interdependency of different strengths, acknowledged and equal, can the power to seek new ways of being in the world generate, as well as the courage and sustenance to act where there are no charters.

(Lorde, 1984, p. 111)

Since writing, the Covid 19 pandemic has further highlighted and exacerbated the problems I outline above. The issue of racism in HE will only become more acute as this crisis continues. While support for “decolonization” has grown somewhat in universities, when Labor Dawn Butler MP suggested “history needs to be decolonized” in schools, Equalities Minister Kemi Badenoch responded that teaching “white privilege” as an “uncontested fact” was “illegal” (Murray, 2020). In fact, it is not, but this debate shows the immediate need to interrogate work on decolonization and antiracism in the academy to challenge reactionary and censorious views such as these. The need for new and creative ways to imagine educational futures is essential. To get to such a place we cannot assume politics follow positionality, nor evade the discomfort of oppositional difference which can form the basis for solidarity in political, social, institutional and interpersonal spaces of higher education.

## Data Availability

Research data are not shared.

## ENDNOTES

^1^
BAME stands for “Black, Asian and Minority Ethnic”. It is a problematic term, conflating as it does issues of race and ethnicity while homogenizing heterogeneous groups who are differentially positioned in British race politics. However, along with BME “Black and Minority Ethnic”, it is used widely in British policy and discourse. I use these terms where and how they are used in the sector, while acknowledging their well‐documented limitations.

^2^
Where applicable I discuss the UK and Britain more widely. Due to differences in national educational provision, in other cases I specify English HEIs.

^3^
These are also unstable and contested terms that are used widely. While there are extensive literature that unpack these terms in great detail, I use them in the more quotidian ways that they are used in the mainstream equalities debates (see below).

^4^
I am aware that any number of other areas of inequality and discrimination could be listed here. I mention disability and gender as areas for which the equalities discourses have most widely documented, but this is not to negate work being done on for example, trans equalities which are now included in the Athena Swan charter mark; sexualities which are addressed in the Stonewall Champions campaign; faith and religion, particularly in relation to the concerns with the Prevent agenda. Action on other areas covered by the Equalities Duty such as age, is largely piecemeal rather than systematic.

^5^
Between 2015 and 2017 a quarter of colleges at Oxford University admitted no Black students (FullFact, 2018). The university figures for 2019 showed some improvement (Coughlan, 2019).

^6^
There was a Race Relations Act in 1965, and 1968. The 1976 Act repealed and superseded the latter.

^7^
I discuss potential limitations of such approaches below.

^8^
These important debates have not been fully taken up by all academic disciplines or engaged with by higher ed institutions. Rather, we see that the meaning of these approaches is highly contested and debates within the university. It is the contested nature of these ideas in institutional contexts that this article explores.

^9^
Recent empirical work shows how scholars are negotiating the global division of academic labor whereby theory is produced in the center, with data gathered at the periphery. They are challenging a passive version of “extraversion” (Hountondji, 1995) in which scholars in the Global South constantly look to Northern sources of authority. Rather they are actively reforming, transforming and adapting knowledge, and also forging new South‐South networks (see e.g., Connell et al., 2018).

^10^
See headlines charging Oxford and Cambridge with unethical investments (The Guardian, 2017) and owning property worth £3.5 billion (The Times, 2018).

^11^
I am not suggesting that decolonization should focus entirely on race and ethnicity nor that disabilities should not be understood in conjunction with the production of racialized positionalities; indeed the entirety of the paper should be read as understood that race and ethnicity are never lived in isolation from other kinds of social category or identification even though they may form a particular kind of collective and communal place of resistance.

^12^
I have also observed numerous moves to “diversify assessment” and within them the same issues arise. The fundamental need to critically analyze content, delivery, outcomes and assessment are often separated. In some cases I have seen stereotypical or even racist views about which students can and will do well with certain kinds of assessments. These kinds of problems remain central to the move to online provision during the current pandemic.

^13^
Again, it is beyond the scope of the paper to go into the debates about terminologies such as decolonial, decolonization, decoloniality and their relationship with postcolonialism or postcoloniality; or the relationship between modernity and coloniality, nor yet their diverse geo‐temporal usages (Ali, 2007). I use the term decolonial and decolonization here as it is used in the mainstream British education discourses for example, “decolonizing the Curriculum” campaigns.
